# Cerebral Amyloid Angiopathy and Parenchymal Amyloid Deposition in Transgenic Mice Expressing the Danish Mutant Form of Human BRI_2_

**DOI:** 10.1111/j.1750-3639.2008.00164.x

**Published:** 2009-01

**Authors:** Ruben Vidal, Ana G Barbeito, Leticia Miravalle, Bernardino Ghetti

**Affiliations:** Department of Pathology and Laboratory Medicine, the Indiana Alzheimer Disease Center, Indiana University School of MedicineIndianapolis, Indiana

**Keywords:** AD, amyloid, CAA, FBD, FDD, neurodegeneration

## Abstract

Familial Danish dementia (FDD) is an autosomal dominant neurodegenerative disease clinically characterized by the presence of cataracts, hearing impairment, cerebellar ataxia and dementia. Neuropathologically, FDD is characterized by the presence of widespread cerebral amyloid angiopathy (CAA), parenchymal amyloid deposition and neurofibrillary tangles. FDD is caused by a 10-nucleotide duplication-insertion in the *BRI_2_* gene that generates a larger-than-normal precursor protein, of which the Danish amyloid subunit (ADan) comprises the last 34 amino acids. Here, we describe a transgenic mouse model for FDD (Tg-FDD) in which the mouse *Prnp* (prion protein) promoter drives the expression of the Danish mutant form of human *BRI_2_*. The main neuropathological findings in Tg-FDD mice are the presence of widespread CAA and parenchymal deposition of ADan. In addition, we observe the presence of amyloid-associated gliosis, an inflammatory response and deposition of oligomeric ADan. As the animals aged, they showed abnormal grooming behavior, an arched back, and walked with a wide-based gait and shorter steps. This mouse model may give insights on the pathogenesis of FDD and will prove useful for the development of therapeutics. Moreover, the study of Tg-FDD mice may offer new insights into the role of amyloid in neurodegeneration in other disorders, including Alzheimer disease.

## INTRODUCTION

Familial Danish dementia (FDD) was first described in members of a single Danish family in the Djursland peninsula (31). The disease was originally named heredopathia ophthalmo-oto-encephalica because of the presence of cataracts, hearing problems and neurological disease (30, 31). Gradual loss of vision, caused by posterior sub-capsular cataract and retinal neovascularization, seems to be the first symptom of the disease which starts before the age of 30. Impaired hearing tends to appear 10–20 years later. Ataxia of a cerebellar type starts after the age of 40. Patients develop slurred speech as well as staggering and swaying gait. Paranoid psychosis usually develops after the age of 50, followed by dementia. Most patients die within the sixth and seventh decade of life (30, 31).

Neuropathologically, FDD is characterized by the presence of cerebral amyloid angiopathy (CAA) in vessels of the retina and leptomeninges as well as in vessels of the gray and white-matter of the central nervous system (CNS) (15, 26, 34). Although CAA is one of the most prominent features of the disease, the incidence of cerebral hemorrhage is rare. All regions of the hippocampus show extensive diffuse amyloid peptide deposition with the presence of large (60–120 µm) cotton-wool-like plaques, abnormal neurites, that are found in the vicinity of blood vessels with amyloid, and neurofibrillary tangles (NFTs) composed of tau paired helical filaments (15, 34). Western blot analysis of the insoluble tau isolated from a patient with FDD showed a pattern similar to that seen in cases of familial British dementia (FBD) (33) and Alzheimer disease (AD) (14, 15). Molecular genetic analysis in patients with FDD revealed the presence of a 10-nucleotide duplication insertion (*BRI_2_795-796InsTTTAATTTGT*) in the 3’-end of the coding region of the *BRI_2_* gene (also known as *ITM2B*(7)), located on the long arm of chromosome 13 (33, 34). The wild-type *BRI_2_* gene encodes a 266 amino acids type-II single transmembrane domain protein of unknown biological function (35). The mutation in *BRI_2_* causes a frame-shift in the BRI_2_ sequence, generating the Danish amyloid precursor protein of 277 amino acids (ADanPP), of which the ∼4 kDa Danish amyloid subunit (designated ADan) comprises the last 34 amino acids (34). Furin and other subtilisin-like pro-protein convertases (PCs) (24) can process both the normal and mutated precursor protein to produce the C-terminal fragments of 23 and 34 amino acids, respectively (17, 18). In addition, all cases of FDD examined so far show the presence of amyloid β (Aβ) deposited in combination with ADan or alone in blood vessels or brain parenchyma (15, 34). The signifi cance of the co-deposition of both types of amyloid is not clear, although Aβ has been found co-deposited with other cerebral amyloids such as cystatin C (37) and prion protein (3).

Herein, we report the generation and neuropathological characterization of a transgenic animal model for FDD (Tg-FDD) in which the Danish mutant form of human *BRI_2_* is expressed under the control of the mouse prion protein promoter. Tg-FDD mice show significant vascular amyloid deposition, parenchymal ADan deposition, amyloid associated gliosis, intracellular and extracellular deposition of oligomeric forms of ADan as well as tau immunoreactive deposits in neuropil. Tg-FDD mice may be considered as a new model of cerebral amyloidosis, which may be useful in further elucidating the pathogenesis of FDD and for testing of diagnostic and therapeutic strategies.

## MATERIAL AND METHODS

### Construction of the MoPrP-*BRI_2_795InsTTTAATTTGT* transgene and generation of transgenic mice

The 10-nucleotide duplication (TTTAATTTGT) (34) was introduced in the *BRI_2_* complementary DNA (cDNA) sequence by polymerase chain reaction (PCR), and the resulting *BRI_2_795InsTTTAATTTGT* cDNA sequence was PCR-amplified using oligonucleotide primers containing an XhoI site, a Kozak consensus sequence and a stop codon. The *BRI_2_795InsTTT AATTTGT* cDNA was inserted into the previously described pBS/MoPrP.Xho vector (2) and the orientation verified by DNA sequencing. The resulting construct was linearized and gel-purified prior to injection into hybrid C3HeB/FeJ mouse embryos. Transgenic lines were established using standard methods at the Indiana University Transgenic and Knock-out Mouse Core Facility. Lines were crossed to non-transgenic C57BL/6J mice for over 10 generations and maintained by crossing transgenic animals to non-transgenic C57BL/6J mice. For genotyping, DNA was extracted from tail clips by proteinase K digestion followed by ethanol precipitation. The transgene was detected by PCR amplification of a 372-basic pair (bp) product using a forward primer (5’-GAT GCC CCA GCT GCT CTC TAC CAG-3’) and a reverse primer (5’-GTA AGT TTC CTT GTC ATG AC-3’) located in the human *BRI_2_* cDNA sequence (33). The care and use of animals in this study were in accordance with institutional guidelines.

### Antibodies and generation of recombinant proteins

Polyclonal antibodies (Abs) were raised in rabbits using synthetic peptides coupled to keyhole limpet hemocyanin through a C-terminal Cys as immunogen. The synthetic peptides were homologous to residues 23–34 (FNLFLNSQEKHYC) of the ADan amyloid peptide (34) (Ab 1700) and residues 24–34 (RTVKKNIIEENC) of the Bri amyloid peptide (ABri) (33) (Ab 1705). The presence of specific antibodies was tested by enzyme-linked immunosorbent assay and dot blot analysis. Commercial polyclonal Abs against glial fibrillary acidic protein (GFAP) (Dako, Carpinteria, CA, USA) for the detection of astrocytes, P-component (Dako) and oligomer-specific antibody (A11) (Invitrogen, Carlsbad, CA, USA) were used, as were monoclonal Abs against apolipoprotein E (ApoE) (3D12, Accurate, Westbury, NY, USA), alpha smooth muscle actin (1A4, Dako), microtubule associated protein tau phosphorylated at Ser202/Thr205 (AT8, Pierce Biotechnology, Rockford, IL, USA), keratan sulfate (5D4, Seikagaku Kogyo, Japan) for the detection of activated microglia, DNA-binding neuron-specific protein NeuN (A60, Chemicon, Temecula, CA, USA) and the Aβ protein clone 10D5 (Elan Corporation, San Francisco, CA, USA) and clone 4G8 (SIGNET, Dedham, MA, USA). For the generation of recombinant ADan peptides, cDNA sequences containing the coding sequences corresponding to amino acids 1–34 of the ADan polypeptide (ADan 1–34), 3–34 (ADan 3–34) and 1–22 (ADan 1–22) (34) were introduced into the Glutathione S-transferase (GST) Gene Fusion Vector pGEX (GE Healthcare Bio-Sciences Corp, Piscataway, NJ, USA) downstream from, and in frame with, the sequence encoding GST. Recombinant polypeptides were expressed by transformed BL21 *Escherichia coli* strains (Invitrogen Corp, Carlsbad, CA, USA) after induction with isopropyl-beta-D-thiogalactopyranoside (Sigma-Aldrich, St. Louis, MO, USA). Cells were disrupted by sonication, and soluble homogenates were used for western blot analysis.

### Histological and immunohistochemical studies

Mice were anesthetized and transcardially perfused with cold 0.9% saline. After perfusion, the animals were decapitated; the skulls opened and the brains removed and kept at 4°C in 4% paraformaldehyde in 0.1 M phosphate buffer, pH 7.2. Eight-micrometer-thick sections were stained with the Hematoxylin-Eosin (H&E) and thioflavin S (ThS) methods. Immunohistochemical labeling was carried out following published protocols (1). For immunohistochemistry, sections were incubated overnight at 4°C with the primary antibodies. Immunostaining was visualized using the avidin-biotin system (Vectastain; Vector Laboratories, Burlingame, CA, USA) and 3,3’-diaminobenzidine (Sigma) as the chromogen. The sections were counterstained with cresyl violet or H&E. Co-localization experiments were done by double-labeling immunofluorescence. Antigen retrieval was performed in a microwave oven in 0.01 M sodium citrate (pH 6.0). Sections were permeabilized in phosphate buffered saline (PBS) containing 0.3% Triton X-100 and unspecific binding was blocked in 10% goat serum, 2% bovine serum albumin, 0.1% Triton X-100 diluted in PBS for 1 h at room temperature. Primary Abs were incubated overnight at 4°C followed by appropriate secondary antibodies conjugated with Alexa-Fluor_488_and Alexa-Fluor_594_ (Molecular Probes Inc, Eugene, OR, USA). Sections were then washed three times in PBS, mounted with ProLong anti-fade (Molecular Probes Inc) and then viewed under a Leica DM4000B microscope with fluorescence attachment and digital camera. Fluorescence images were acquired with a Zeiss LSM 510 confocal microscope equipped with argon and helium/neon lasers using an inverted 63× NA 1.2 water-immersion objective.

### Immunoblot analysis

Brain tissue was homogenized in 10 vol of 50 mM Tris (pH 8,0), 150 mM NaCl and 5 mM EDTA containing a proteinase inhibitor cocktail (Complete, Roche Molecular Biochemicals, Indianapolis, IN, USA). Amyloid was extracted by incubation with 99 % formic acid (Sigma) for 2 h at room temperature and the formic acid-soluble material was dried under a N_2_ atmosphere as described (34). Amyloid peptides and recombinant proteins were run on a 16% Tris-Tricine SDS-PAGE. Proteins were electro-transferred onto polyvinylidene difluoride membranes (Immobilon-P, Millipore Corporation, Billerica, MA, USA) using 10 mM 3-cycloexylamino-1-propanesulfonic acid buffer, pH 11, containing 10% methanol (Sigma). The membranes were blocked with 5% non-fat dry milk in 10 mM phosphate buffer, 137 mM NaCl, 2.7 mM KCl (PBS, Sigma) pH 7.4 with 0.1% Tween-20 (PBS-T) overnight and then incubated for 2 h at room temperature with Ab 1700. Horseradish peroxidase-conjugated goat anti-rabbit (Amersham, Piscataway, NJ, USA) was used as the second Ab at a dilution of 1:5000 in PBS-T. Immunoblots were visualized by chemiluminescence (Amersham) according to the manufacturer's specifications.

## RESULTS

### Generation of transgenic mice expressing the Danish mutant form of *BRI_2_*(Tg-FDD)

**Figure 1:**
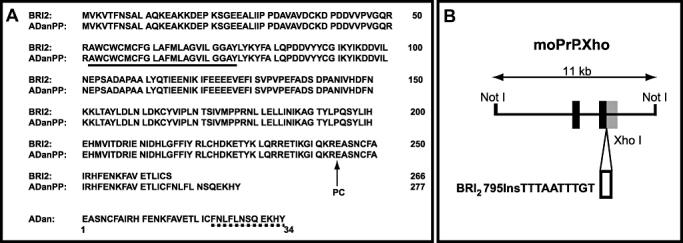
*Amino acid sequence of ADanPP,ADanand construction of the expression vector*. A. The wild-type BRI_2_ polypeptide consists of 266 amino acids. The Danish BRI_2_ mutant polypeptide (ADanPP) has 277 amino acids. The arrow indicates the pro-protein convertase (PC) cleavage site that releases the ADan peptide. The putative transmembrane domain is underlined. The ADan sequence consists of the last 34 amino acids of the mutant protein. The sequence recognized by polyclonal antibody 1700 is underlined. B. Expression plasmid moPrP.Xho containing a complementary DNA carrying the Danish mutant *BRI_2_* sequence. Black boxes, non-coding 5’ sequences; gray box, non-coding 3’ sequences; open box, Danish mutant *BRI_2_* -coding sequence.

**Figure 2:**
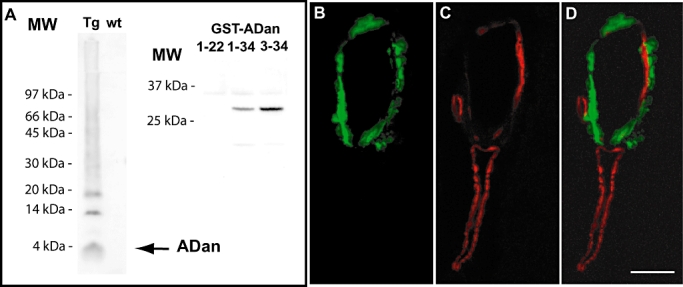
*ADanamyloid peptides in transgenic-Familial Danish dementia (Tg-FDD) mice*. A. Western blot using polyclonal antibody (Ab) 1700 (anti-ADan) of amyloid isolated from Tg-FDD mice (Tg) and wild-type control mice (wt). ADan monomers (arrow), trimers and tetramers are seen in the formic acid soluble fraction of Tg-FDD mice. Ab 1700 recognizes recombinant ADan peptides that contain the full-length ADan sequence (1–34) or a sequence starting at position 3 of ADan (3–34) fused to Glutathione S-transferase (GST), but not a fusion protein containing only the first 22 amino acids of the ADan peptide (1–22) (Inset). B–D. Laser scanning confocal microscopy of leptomeningeal vessels. Immunolabeling for ADan (B) and vascular alpha smooth muscle actin (C) in a meningeal vessel. Loss of smooth muscle cells in the amyloid-rich regions of the vessel is observed in the merge image (D) in sections from an 18-month-old homozygous Tg-FDD mouse. Immunofluorescence using Ab 1700 (B) and anti-alpha smooth muscle actin (C). Scale bar: B–D, 20 µm.

We generated transgenic mice by microinjecting a construct containing a cDNA encoding the Danish mutant form of human *BRI_2_* (Figure 1A) to oocytes of C3HeB/FeJ hybrids. Five independent transgenic lines were obtained, three of which produced stable lines of mice that express the transgene. Expression of the transgene in the CNS was driven by the mouse prion protein (*moPrnp*) promoter (2) (Figure 1B). The presence of the transgene was confirmed by PCR amplification of a 372-bp fragment, specific for the human *BRI_2_* cDNA sequence (33). All three lines developed accumulation of amyloid in the brain with similar regional deposition and were studied together. ADan amyloid was detectable in animals older than 7–8 month of age by western blot analysis. ADan was present in the formic acid soluble fraction mostly as a ∼4 kDa monomer (Figure 2A, lane 1, see arrow). ADan polymers, in particular trimers and tetramers, were also seen by western blot analysis using Ab 1700. No immunoreactivity was seen in control mice (lane 2) or using Ab 1705, against the ABri peptide (not shown). The specificity of Ab 1700 was confirmed by western blot analysis using recombinant proteins (Figure 2A, inset).

As the animals aged, Tg-FDD mice displayed an abnormal grooming behavior. One-year-old transgenic mice showed an arched back and walked with a wide-based gait and shorter steps. Feet clasping was observed upon suspension of the mice by their tails. Transgenic mice would flex their front and hind limbs inward, with paws clasped together and drawn in toward the body. In contrast, wild-type mice demonstrate normal limb posture when suspended by their tails.

### Vascular amyloid deposition in Tg-FDD mice

**Figure 3:**
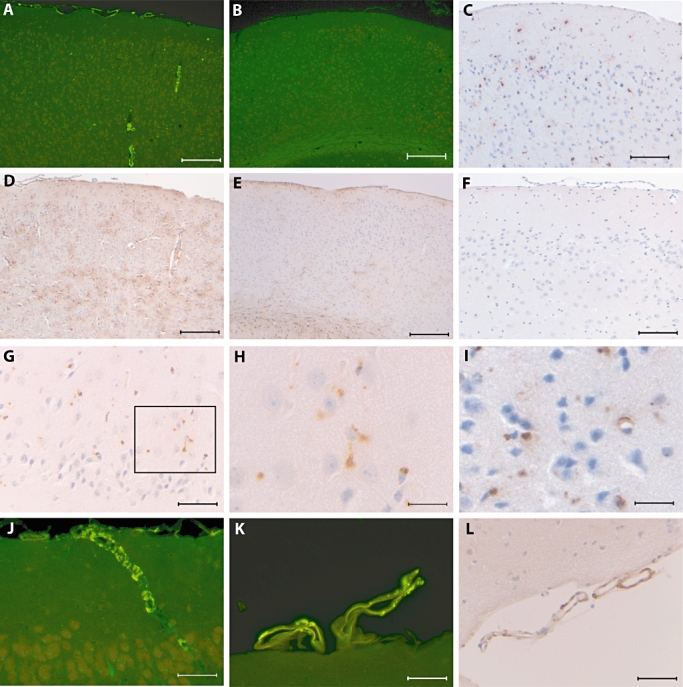
*Vascular and parenchymal amyloid deposition in the cerebral cortex*. A. All cerebral cortical layers of transgenic-Familial Danish dementia (Tg-FDD) mice show amyloid deposition and an increase in the number of reactive astrocytes (D). Leptomeningeal and parenchymal amyloid deposits were immunostained using polyclonal antibody (Ab) 1700 (C,G), which also stained the subpial region of the cerebral cortex. H.Enlargement of the area indicated in (G) shows the presence of concentrated (dot-like) and diffuse parenchymal deposits. I. High magnification photograph showing parenchymal and some vascular deposits. Amyloid deposition was seen in penetrating cortical vessels (J) and in leptomeninges (K). L.Leptomeninges immunolabeled using Ab 1700. Sections were from a homozygous 14 (G,H), homozygous 18 (C,I,J), and heterozygous 21-(A,D,K,L)-month-old Tg-FDD mice and a 21-month-old control mouse (B,E,F). Thioflavine-S (A,B,J,K). Immunohistochemistry using Ab 1700 (C,F–I,L) and anti- glial fibrillary acidic protein (D,E). Scale bars: A,B,D,E, 200 µm; C,F, 100 µm; G,J–L, 50 µm; H,I, 20 µm.

**Figure 4:**
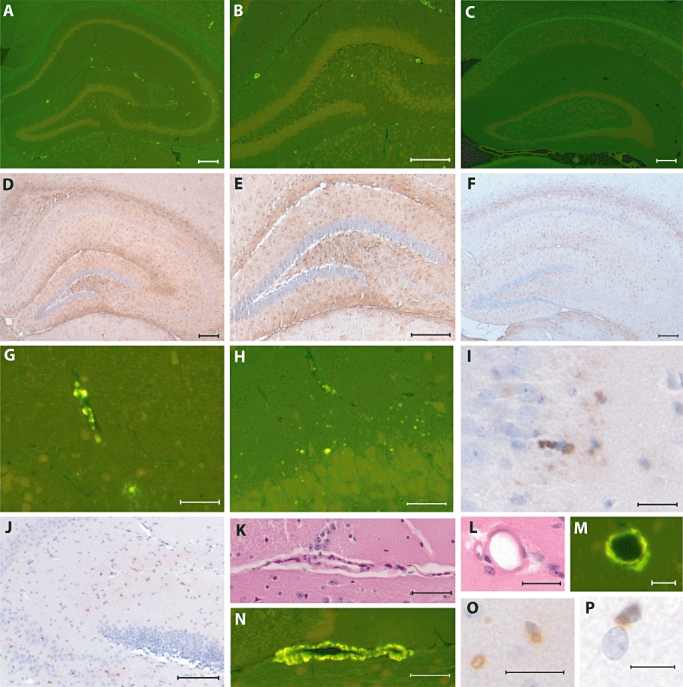
*Vascular and parenchymal amyloid deposition in the hippocampus*. Amyloid deposition is seen throughout the hippocampal formation (A,B) of transgenic-Familial Danish dementia (Tg-FDD) mice and is accompanied by an increase in the number of reactive astrocytes (D,E). Age-matched non-transgenic littermates showed none of these neuron-pathological changes (C,F). Vascular amyloid deposition is present in the walls of large and medium size vessels (A,B,G,H,M) and in the walls of vessels of the hippocampal fissure (B,N). Vessels containing amyloid show thickened, eosinophilic walls (K,L). Thioflavin S fluorescent small, punctate deposits, ∼3 µm in diameter, are seen in the hippocampus (H). Anti-ADan-immunopositive structures outline the hippocampus (J), which are either well defined and plaque-like or diffuse (I,J,O). Some polyclonal antibody (Ab) 1700-immunopositive structures appear intracellular (I,O,P). Sections were from a homozygous 14 (G,O,P), homozygous 18 (I,J), heterozygous 18 (K–N), and heterozygous 21-(A,B,D,E,H)-month-old Tg-FDD mice and a 21-month-old control mouse (C,F). Thioflavine S (A–C,G,H,M,N). Immunohistochemistry using Ab 1700 (I,J,O,P) and anti-glial fibrillary acidic protein (D–F). Hematoxylin-Eosin (K,L). Scale bars: A-F, 200 µm; G,H,J–L,N, 50 µm; I,M,O,P, 20 µm.

**Figure 5:**
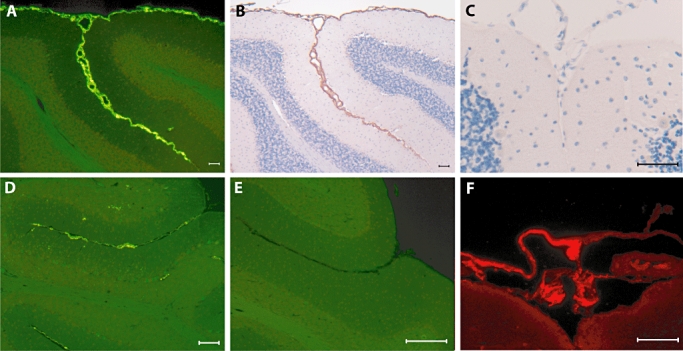
*Cerebellar leptomeningeal amyloid deposition*. Amyloid deposition is observed in pial (leptomeningeal) vessels of the cerebellum (A,D) of transgenic-Familial Danish dementia (Tg-FDD) mice but not in age-matched non-transgenic littermates (E). Antibodies against ADan immunolabeled leptomeningeal and cortical blood vessels, and the subpial region of the cerebellar cortex of Tg-FDD mice (B). No immunoreactivity is seen in non-transgenic littermates (C). F. Leptomeninges are immunolabeled using polyclonal antibodies (Abs) against ApoE. Sections were from a heterozygous 9 (F), homozygous 18 (A,B), and heterozygous 21-(D)-month-old Tg-FDD mice and a 21-month-old control mouse (C,E). Thioflavine S (A,D,E). Immunohistochemistry using Ab 1700 (B,C) and anti-ApoE (F). Scale bars: A,B,D,E, 200 µm; C,F, 50 µm.

Brains from 34 heterozygous and homozygous mice and 12 age-matched non-transgenic littermate controls (1–24 months old) were neuropathologically examined. Between 1 and 6 months of age, no obvious pathology was detected. At ∼7 months of age, transgenic animals consistently began to exhibit CAA primarily in pial (leptomeningeal) cerebellar vessels. As the animals aged, (≥8–9 months), large and medium-sized parenchymal and penetrating vessels of the cerebrum (neocortex, hippocampus, thalamus and olfactory bulb), the brain stem and the spinal cord also showed amyloid angiopathy (Figures 3–5). CAA was present in all transgenic mice studied and amyloid deposition was seen to increase with age. As in patients with CAA (36), individual vessels had a varying extent of amyloid deposition, sometimes seen in the form of focal globular clumps (Figure 3K). In the hippocampus, vascular amyloid deposition was seen in the walls of large and medium size vessels (Figure 4G,H) and in the wall of vessels of the hippocampal fissure (Figure 4B,N). Vessels affected by CAA showed thickened, eosinophilic walls (Figure 4K–N). Abs against the ADan peptide immunolabeled vascular deposits in Tg-FDD mice but did not label vessels of non-transgenic littermates (Figures 3–5). Parenchymal vessels of the spinal cord that contained amyloid deposits were also immunolabeled by Ab 1700. Vascular deposits were also immunostained using Abs against P-component (not shown) and ApoE (Figure 5F). Preliminary attempts to identify Aβ deposits were unsuccessful. Non-transgenic littermates showed none of these neuropathological changes.

Accumulation of ADan amyloid in vessels was accompanied by a loss of smooth muscle cells (SMCs) in the vessel wall as shown by the depletion of vascular SMC α-actin (Figure 2B–D) and a decrease in the number of SMC nuclei. Vessels that were not affected by amyloid deposition showed a continuous rim of SMCs. Despite the massive occurrence of amyloid angiopathy in Tg-FDD mice, in none of the old mice (21 months) we analyzed did we detect hemorrhages.

### Parenchymal accumulation of ADan in Tg-FDD mice

**Figure 6:**
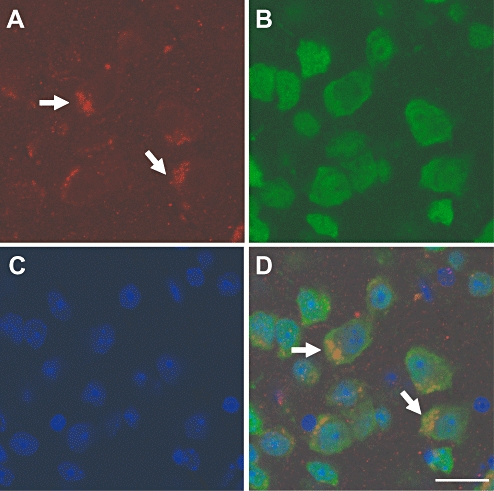
*Co-localization of ADanand NeuN immunoreactivities*. Laser scanning confocal microscopic analysis of cerebral cortex of an 18-month-old homozygous transgenic-Familial Danish dementia (Tg-FDD) mouse showing the localization of polyclonal antibody 1700-immunopositive (arrows) deposits (A) in neurons expressing the neuronal marker NeuN (B). Nuclear (DAPI) staining (C) and merged image (D). Scale bar: 20 µm.

Parenchymal amyloid deposition was seen in brain areas affected by CAA (Figures 3 and 4) with the exception of the cerebellum, where it was relatively uncommon to observe the presence of ThS fluorescent material that was not associated with vessels (Figure 5). In the cerebral cortex we observed the presence of ThS-fluorescent deposits of ∼11 µm in diameter and some small punctate deposits of ∼3 µm in diameter (Figure 3A). These deposits were immunolabeled by Ab 1700. Widespread diffuse deposition of ADan was also observed between the same regions (Figure 3C,G–I). Immunoreactivity was not seen in non-transgenic littermates (Figure 3F) or using Ab 1705 (not shown). Some of the Ab 1700-immunopositive parenchymal deposits appeared to be within oligodendrocyes (Figure 3G–I). Double-labeling of neurons with Abs against NeuN and Ab 1700 showed co-localization of both immunoractivities (Figure 6). Abs against GFAP and activated microglia failed to reveal co-localization when used in combination with Ab 1700.

Parenchymal amyloid deposition was also seen throughout the hippocampus and was most prominent in the CA3 and CA2 regions and the hilus (Figure 4A,B). Amyloid deposits were seen as small plaques (7–10 µm in diameter) that sometimes showed a weak ThS fluorescent surrounding (Figure 4G,H) and in the form of punctate structures (Figure 4H). Ab 1700 immunolabeled amyloid deposits (Figure 4I,J,O,P), and also showed the presence of abundant ADan diffuse deposits (Figure 4I,J). As in the cerebral cortex, some of the immunopositive deposits detected using Ab 1700 appeared to be intracellular (Figure 4I,O,P). Furthermore, Ab 1700 revealed the presence of diffuse parenchymal deposition of ADan in the spinal cord (not shown). Analysis of H&E-stained sections occasionally revealed neurons with a pyknotic appearance, in particular in cortical layer II of the cerebral cortex, and neuronal shrinkage and loss of Purkinje cells in the cerebellum. Non-transgenic littermates showed none of these neuropathological changes.

### Amyloid accumulation is associated with an astrocytic response and microglial activation

**Figure 7:**
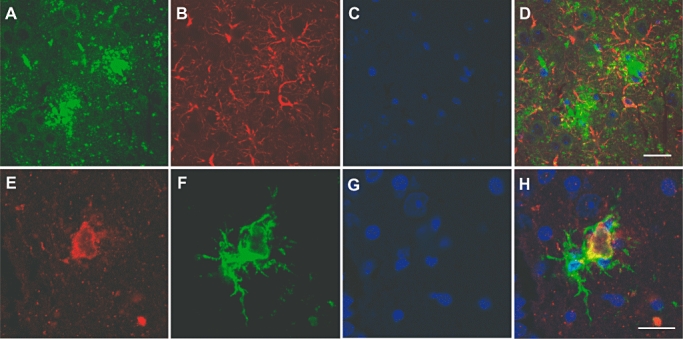
*Close association between amyloid deposits, astrocytes and microglia*. Laser scanning confocal microscopic analysis of hippocampal sections of a homozygous 18-month-old transgenic-Familial Danish dementia mouse showing the localization of polyclonal antibody 1700-immunopositive deposits (A,E), glial fibrillary acidic protein-positive reactive astrocytes (B), keratan sulfate-positive-activated microglia (F), nuclear (DAPI) staining (C,G) and merged images (D,H). Scale bars: A–H, 20 µm.

Analysis of H&E-stained cerebral cortical sections of Tg-FDD mice revealed the presence of a severe gliosis. Numerous GFAP-positive reactive astrocytes were observed throughout all neocortical areas showing amyloid deposition (Figure 3D). Only a weak astrocyte immunostaining was observed in all examined brain regions of age-matched wild-type mice (Figure 3E). The presence of numerous GFAP-positive reactive astrocytes was also noted in the hippocampus of Tg-FDD mice (Figure 4D,E) but not in non-transgenic littermates (Figure 4F). In general, activated astrocytes in the hippocampus were seen in areas affected by ADan amyloid deposition (Figure 7A–D) but not in areas devoid of amyloid. In addition to astrocytes, we observed 5D4-immunopositive microglia located in the close vicinity of the vascular and parenchymal amyloid deposits. Microglial cell processes were seen surrounding amyloid deposits (Figure 7E–H), but were not observed in association with diffuse deposits.

### Deposition of oligomeric forms of ADan and presence of abnormal tau deposits in Tg-FDD mice

**Figure 8:**
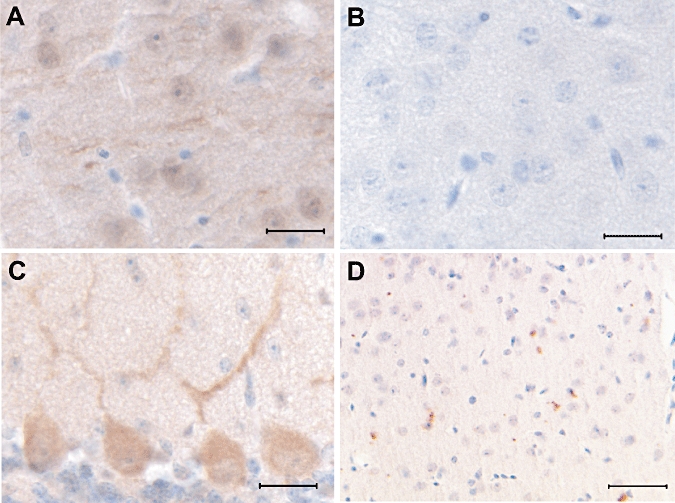
*Oligomeric forms of ADanand tau deposition in transgenic-Familial Danish dementia (Tg-FDD) mice*. Oligomeric forms of ADan are detected in the cerebral frontal cortex (A) and in cerebellar purkinje cells (C). No immunoreactivity is seen in sections of the cerebral frontal cortex of a control mouse (B). D.Hyperphosphorylated tau-positive structures surrounding amyloid deposits in the frontal cortex. Sections were from a homozygous 18-month-old Tg-FDD mouse (A,C,D) and a 21-month-old control mouse (B). Immunohistochemistry was done using polyclonal antibody (Ab) A11 (A–C) and Ab AT8 (D). Scale bars: A–C, 20 µm; D, 50 µm.

A robust immunostaining was observed throughout the cerebrum (Figure 8A) using Ab A11, which recognizes Aβ oligomers and oligomers of other proteins involved in neurodegenerative diseases (16). Immunostaining was seen both intra- and extra-cellularly in Tg-FDD mice but not in wild-type controls (Figure 8B). In the cerebellum, Ab A11 immunostaining was seen in cell bodies and dendrites of Purkinje cells (Figure 8C). Staining of adjacent sections with ThS revealed lack of co-localization of A11 immunostaining and ThS fluorescence, strongly suggesting that the deposits recognized by Ab A11 represent ADan oligomers. Immunohistochemistry using Ab AT8 revealed the presence of numerous tau-immunopositive deposits throughout the neuropil of the cerebral cortex and hippocampus (Figure 8D). No tangle formation was seen by ThS fluorescence.

## DISCUSSION

We generated transgenic mice expressing the Danish mutant form of *BRI_2_*(34). As in FDD, CAA is one of the main neuropathological findings in this model with vascular amyloid leading to a loss of SMCs. We also observed the presence of parenchymal (mostly diffuse) deposition of ADan and accumulation of oligomeric forms of the peptide. Some ThS fluorescent parenchymal amyloid deposits were also seen in the cortex and hippocampus. In Tg-FDD mice, amyloid burden appears to increase with age implying a progressive ADan deposition that exceeds its clearance. As in FDD, amyloid deposition leads to a neuroninflammatory response (15). Although we found tau-positive structures, we did not detect NFTs. Thus, Tg-FDD mice may be considered a new model of cerebral amyloidosis.

One of the main neuropathological features of FDD is the presence of widespread CAA (15, 34). In Tg-FDD mice, expression of the Danish mutant form of *BRI_2_* driven by the *moPrnp* promoter is sufficient for the development of CAA. CAA and parenchymal plaques have been also observed when the expression of the mutant *BRI_2_* sequence is driven by the Syrian hamster *Prnp* promoter (6). Thus, the presence of CAA and the localized loss of SMCs seen in the Tg-FDD mouse model strongly suggest that neural cells are the source of the cerebrovascular amyloid in FDD. In addition, it denotes the vasculotropic nature of the ADan peptide. The solubility properties of ADan may permit this molecule to diffuse, resulting in ADan being deposited around leptomeningeal vessels, as it has been suggested for Aβ1-40 (38). Our data also suggest that loss of SMCs does not require intracellular ADan production and that it may be induced *in vivo* by extracellular amyloid peptides, as it has been suggested for transgenic mice models expressing mutant forms of the amyloid β precursor protein (AβPP) (8, 13, 22, 32). Although CAA is considered an important cause of intracerebral hemorrhage as well as ischemic stroke (27), we have not observed any evidence of cerebral hemorrhages in Tg-FDD mice. Alternatively, vascular deposition of ADan may cause problems with the function of affected vessels. As described by Christie et al. (5), amyloid may interfere with the function of the vessel by: (i) presenting a mechanical obstacle to vessel dilation, rendering the vessel wall relatively rigid; (ii) causing a physical separation of adjacent SMCs, which may disrupt contraction dependent on their coordinated action; and (iii) having a toxic effect on SMCs that interfere with the ability of the vessel to dilate appropriately. Future *in vivo* and *in vitro* studies using cerebrovascular cells may explain the role of ADan peptides in SMC degeneration and the possible contribution of CAA to the pathology seen in FDD.

In the Tg-FDD mouse model, the deposition of vascular amyloid was accompanied by large increases in the number of reactive astrocytes and activated microglia. Neuroinflammation associated with amyloid deposition has been observed in patients with FDD (15, 29), AD (28) and in mutant AβPP transgenic mice (13, 22). Whether the inflammatory response, characterized by the released of potentially neurotoxic substances and cytokines, may cause neurodegenerative changes in neurons, or may have a neuroprotective role by stimulating the clearance of the amyloid deposits, remains to be determined (23). Importantly, treatments designed to reduce CAA-induced neuroinflammation appear to have lessened the dementia in affected individuals (12, 25). Moreover, the reduction of the cerebral microvascular deposition of Aβ in an animal model expressing a human Swedish/Dutch/Iowa mutant AβPP has been shown to diminish regional neuroinflammation (21).

As seen in FDD patients (34), Tg-FDD mice showed presence of abundant diffuse ADan deposits in the cerebral parenchyma. In addition, we observed the presence of small, dot-like, fluorescent ThS structures. Larger plaque-like amyloid deposits were sporadically observed, and whether these deposits are related to blood vessels remains to be determined. These plaque-like amyloid deposits were seen associated with GFAP-labeled astrocytes and activated microglia. This finding suggests that ADan amyloid deposition in Tg-FDD may cause the activation of an inflammatory process, similarly to what has been reported in animal models of AD (9, 11, 12, 21, 23, 25). We also found intracellular Ab 1700-immunoreactivity in close proximity to the cell nucleus in Tg-FDD mice. This finding may reflect intracellular accumulation of the amyloid peptide or the precursor protein as the residues recognized by Ab 1700 are present in both the ADan peptide and the ADanPP sequences (34). Processing of the precursor protein by PCs (17, 18) and by ADAM 10 and SPPL2a/b (19) seems to be initiated upon the arrival of the protein to the Golgi (4). After processing, amino-terminal proteolytic fragments accumulate intracellularly and the carboxyl-terminal processing products are secreted via a regulated secretory pathway (4). *In vitro* studies using transfected cell lines have suggested that while the ABri peptide found in patients with FBD (33) is detected both intracellularly and in the medium, the ADan peptide accumulates predominantly in intracellular compartments (18). Whether intracellular accumulation of ADan peptides results in neuronal dysfunction leading to neurodegeneration remains to be seen. In contrast to Tg-FDD mice, transgenic mice expressing a BRI_2_protein–Aβ_42_ fusion construct that generates only Aβ_42_ in the brain show little evidence of intraneuronal Aβ accumulation (20).

The presence of ADan-oligomers detected with Ab A11 is highly indicative that a soluble pool (intracellular and extracellular) of ADan may play a role in neurodegeneration. *In vitro* studies have shown that fresh solutions containing soluble oligomers of ADan are more toxic to neuronal cells than aged solutions containing insoluble aggregates (10). Interestingly, the study of Aβ oligomers which do not co-localize with the Aβ fibril plaques in mouse models have shown that Aβ oligomers can impair synaptic function and spatial memory and may contribute to some of the early cognitive dysfunction in AD (16). Thus, the study of oligomeric forms of ADan in Tg-FDD mice may allow us to determine the exact roles of intracellular and extracellular ADan peptides in neuronal toxicity and to investigate whether similar mechanisms of toxicity exist between FDD and AD *in vivo*. Our results suggest that Tg-FDD mice may provide a unique model of cerebral amyloid deposition that will allow drawing parallels with mouse models of AD. Moreover, this model may provide important information on whether amyloid peptides, regardless of their primary amino acid sequence, share common pathogenic mechanisms.
